# Potential toxic effects of titanium oxide (TiO_2_) nanoparticles on the biological, biochemical, and histological aspects of the land snail *Helix aspersa*

**DOI:** 10.1007/s11356-023-27666-y

**Published:** 2023-06-02

**Authors:** Hoda H. Abdel-Azeem, Gamalat Y. Osman, Azza H. Mohamed

**Affiliations:** Department of Zoology, Faculty of Science, Menoufia University, Shebin El-Kom, Egypt

**Keywords:** *Helix aspersa*, Oxidative stress biomarkers, Titanium oxide nanoparticles, Haemocytes, Comet assay

## Abstract

Nanotechnology has come a long way in our lives. However, it maintains some negative effects on the environment. This study aims to use the land snail *Helix aspersa* as a bioindicator. Titanium dioxide nanoparticles (TiO_2_NPs) had been used at 70 and 140 µg/L for two weeks by the spraying method. The oxidative biomarkers, condition index (CI), DNA damage, hemocyte count, and phagocytic activity were estimated. The toxicity of TiO_2_NPs was determined (LC_50_ = 544 µg/L). The exposure to TiO_2_NPs caused a significant reduction of the activities of superoxide dismutase (SOD) and catalase (CAT) in the digestive gland of *Helix aspersa* (the activity of CAT was 3.4 ± 0.1 (*P* = 0.001), SOD was 11 ± 1 (*P* = 0.0002) at concentration 140 µg/L after two weeks). The activity of glutathione peroxidase (GPX) was (1.13 ± 0.01 µ/mg protein at 140 µg/L compared with controls (5.47 ± 0.01 µ/mg protein). The treatment caused DNA damage in the hemocytes (tail DNA % = 8.66 ± 0.02 and tail moment = 52.99 ± 0 at140 µg/L (*P* = 0.002)). In the digestive gland, both tail DNA % and tail moment increased (tail moment = 78.38 ± 0.08 compared with control = 2.29 ± 0.09 (*P* = 0.0001)). The total count of hemocytes significantly decreased after two weeks (the average number was 71 ± 1.5 compared with controls 79 ± 1.1 at 140 µg/L). Furthermore, TiO_2_NPs caused histological alterations in the digestive gland of *Helix aspersa*. It can be concluded that the *Helix aspersa* can be used as environmental pollution bioindicator. A comprehensive evaluation of toxic effects induced by TiO_2_NPs in vivo assays must be investigated.

## Introduction

Although the land snail *Helix aspersa* is considered an agricultural pest, it can be used as an environmental pollution bioindicator. *Helix aspersa* can be affected by and accumulate many metals (Viard et al. 2004; Abdel-Halim et al. 2013). Nanoparticles (NPs) have numerous applications, especially titanium oxide (TiO_2_) which are used in cosmetic products, food additives, and agricultural fertilizers. NPs can reach aquatic ecosystems through the discharge of treated water (Ropers et al. 2017; Lu et al. 2015). Nanotechnology is a prominent field and enhances the quality of our life. However, the negative environmental effects of NPs extend its adversity to human lives (Singh and Prasad 2013; Kaloyianni et al. 2020).

Certain foreign compounds (xenobiotics) can directly mediate the formation of reactive oxygen species (ROS), e.g., superoxide and peroxides, that can cause damage to biomolecules and may affect signaling pathway (Hofer 2021). Moreover, the xenobiotics may also indirectly induce oxidative stress by affecting antioxidants enzymes and non-enzymatic antioxidants. Such alterations can be used as effective biomarkers to study environmental pollution. Reactive oxygen species (ROS) caused cell damage by disrupting DNA, oxidation of lipids, and modifying proteins (Huang et al. 2017). The cells can respond to enzymatic expression (Kovochich et al. 2007; Poletta et al. 2016).

Several authors assessed the toxicity of different nanoparticles on *Helix aspersa* (Abdel-Azeem and Osman 2021 on zinc oxide nanoparticles (ZnONPs); Besnaci et al. 2016a on iron oxide powder (Fe_2_O3) nanoparticles; Khene et al. 2017 on TiO_2_ microparticles). Feidantsis et al. (2020) confirmed the toxicity of both copper oxide nanoparticles (CuO NPs) and ZnO NPs on the land snail *Cornu aspersum* hemocytes, where they caused an increase in ROS production, DNA integrity loss, and changes in the NRRT50 values. Abdel-Halim et al. (2021) confirmed the presence of oxidative stress on the digestive gland and hemolymph of the snail *Helix aspersa* after dermal exposure to TiO_2_NPs. Maroua et al. (2022) evaluated the toxicity of CuO-NPs on the hepatopancreas and kidney of *Helix aspersa* by evaluating the oxidative stress biomarkers (CAT, GPX, GSH, GST, and LPO). Abdel-Halim et al. (2022) recorded DNA damage and histopathological defects in the nucleus, microvilli, mitochondria, and execratory glands of the digestive gland of the snail *Monacha cartusiana* after exposure to zinc oxide nanoparticles.

Besnaci et al. (2016b) recorded histopathological alterations in the hepatopancreas of *Helix aspersa* as a response to three doses of Fe_2_O3NPs such as degeneration and inflammatory infiltrates. Morsy et al. (2022) concluded that silver nanoparticles induced histological changes in the digestive gland of the land snail *Monacha obstructa*. The hepatopancreas of *Helix aspersa* can accumulate and its structure was altered after exposure to xenobiotics and chemicals (Grara et al. 2012; Regoli et al. 2006). The single-cell gel electrophoresis (comet assay) is a detective method for DNA damage after exposure to NPs, ionizing radiation, and chemical compounds in different organisms (Ali et al. 2008).

So, this study aims to explore the ecotoxicity of TiO_2_NPs by using the model *Helix aspersa*. This evaluation can be done by the determination of the oxidative stress biomarkers, such as SOD, CAT, and GPX, as long as DNA damage is induced by comet assay, hematological effects, and histopathological effects on the digestive gland and ovotestis.

## Materials and methods

### Toxicity test

A series of concentrations were prepared. Three replicates with ten snails (4 ± 0.4 g) for each were exposed. After 24 hrs of exposure, dead snails were counted, removed, and LC_50_ was computed by Probit analysis via statistical software of social sciences (IBM SPSS) (IBM Corp. Armonk, NY, USA).

### Experimental design

The gastropod *Helix aspersa* used in the study was acclimatized for experimental conditions, (temperature 22 ± 2 °C, photoperiod 12 h, humidity, 75 to 85%). Ninety snails were used and divided into three groups: The 1^st^ group was unexposed, to be the controls. The 2^nd^ group was exposed to 70 µg/L. And the 3^rd^ group was exposed to 140 µg/L. They were kept in glass boxes and fed lettuce. The exposure period was two weeks. The used concentrations were prepared as the estimated solid nanoparticles were mixed with double distilled water (ddH_2_O) and sonicated (Soniprep 150, MSE) for 10 min to prevent aggregation. The application method was carried out by spraying. Tissue samples were collected at three different time points: after one, three days, and one and two weeks of exposure to determine the condition index, oxidative stress biomarkers, comet assay, and histological examination. Hemolymph was collected after one day of exposure for comet assay and phagocytosis.

### Characterization of TiO_2_NPs

The samples were prepared from the TiO_2_ powder for the transmission electron microscope (TEM) and X-ray diffraction (XRD). A small amount of powder was put in isopropanol and the suspension was left for 10 mins in an ultrasonic bath to avoid forming agglomerations of particles. Two drops of the suspension were placed on a 3-mm diameter carbon-coated copper grid, and the alcohol evaporated completely in air. The samples were photographed with a JEOL JEM-1400 Plus transmission electron microscope in the electron microscope unit at the Faculty of Science, Alexandria University, Elshatby). For recording the XRD pattern, TiO_2_ nanoparticles were coated into a thin film on a cleaned glass substrate.

### Condition index

The condition index (CI) is an index of current organism nutritive and was calculated as follows (Aguirre 1979):

$$\mathrm{CI}=[\mathrm{MW}/(\mathrm{TW}-\mathrm{SW})]\times 100$$

### Oxidative stress markers

#### Catalase (CAT) activity

The CAT assay based on the disappearance of H_2_O_2_, which is caused by the action of catalase and the rate of decrease, is a gauge of the amount of catalase catalyzing the reaction, 2 H_2_O_2_ CAT 2H_2_O + O_2_: one unit of catalase activity decomposes one micromole of H_2_O_2_ per minute at 25C° and pH 7.0 under the specific conditions. The reaction mixture consisted of 0.05 M potassium phosphate pH 7.0 and M H_2_O_2_ in 0.05 M potassium phosphate working buffer (pH 7.0) (Aebi 1984).

#### Glutathione peroxidase (GPX) activity

The determination of this enzyme is based on the formation of GSH from GSSG by the action of GR in presence of NADPH. The activity of the total amount of GPx is based on the measurement of cumene hydroperoxide. One unit of enzyme activity was defined as the amount of enzyme required to oxidize 1 μmol of NADPH per minute. The reagents were potassium phosphate buffer 50 mM, pH 7, EDTA–azide solution was prepared by dissolving 5 mM EDTA and 1 mM azide in the phosphate buffer, 2 mM GSH in the phosphate buffer, 0.25 mM H_2_O_2_, 1.5 mM cumene hydroperoxide in ethanol, 50 U/mL glutathione reductase, and 0.2 mM NADPH in phosphate buffer pH 7 (Flohe and Gunzler 1984).

#### Superoxide dismutase (SOD) activity

Superoxide dismutase is an enzyme that catalyzes the dismutation of superoxide into O_2_ and H_2_O_2_. Tissue was homogenized (5 mL/g) in cold lysis buffer (50 mM potassium phosphate, 0.1 mM EDTA, 0.5% Triton X-100), centrifuged at 12,000 g for 5 min at 4 °C. The supernatant was used for total SOD assay according to EnzyChromTM Superoxide Dismutase Assay Kit (ESOD-100) (Sahin et al. 2020).

### Comet assay

Single-cell gel electrophoresis (comet assay) was performed in the tissues according to the method of (Singh et al. 1988). The tissue was minced using small dissecting scissors into very small pieces in a chilled buffer consisting of 0.075 M NaCl and 0.024 M Na2EDTA. Then, they were homogenized via a homogenizer. Cells suspension was centrifuged at 700 × *g* for 10 min at 4 °C, followed by resuspension in a cold buffer, and a pellet was obtained. Cells were mixed with molten LM agarose (low-melting point agarose) followed by the spread of the mixture over a frosted slide. The slides were placed in lysis solution for 60 min followed by electrophoresis at a high pH value of 13. Then, the slides were immersed in a neutralization buffer for 15 min. Samples were dried, stained with ethidium bromide, and viewed by an epifluorescence microscope. A Leitz Orthoplan epifluorescence microscope was used to perform image analysis, which was equipped with an excitation filter of 515–560 nm and a barrier filter of 590 nm. The microscope was connected to a computer-based image analysis system (Comet Assay V software, Perspective Instruments). To score comet, (50–100) randomly selected cells per slide were used. DNA damage was evaluated as tail length, %tail DNA, and tail moment.

### Histological studies

Histological examinations were carried out at the end of the experiment (14 days). The digestive gland was dissected out and immediately fixed in Bouin᾿s fluid. After 24 h of fixation, samples were then dehydrated through a series of alcohols and cleared in xylene. Paraffin wax blocks were made (Romeis 1989). Then, sections (5–7 μm thickness) were cut and stained with hematoxylin and eosin (Mayer’s H and E). Staining was followed by a good wash with tap water. Histological sections were photographed using a photo-automated camera.

### Total and differential hemocyte counts

Hemolymph was collected. When the snail retracted into its shell, a drop of hemolymph was extruded through the hemal pore with a micropipette. Hemolymph (10 µL) was directly dropped in a hemocytometer. For the total number of living hemocytes and the number of hemocytes in each cell, the population was identified according to (Van der Knaap et al. 1993; Suljevic et al. 2019). Nauber’s hemocytometer was used for the analysis of the number of hemocytes. Giemsa stain (10 µL) was added to 40 µL of hemolymph (0.8 dilution factor), stirred gently, and left for 5 min at room temperature to dye the hemocytes. For counting, 10 µL of diluted hemolymph was used. The number of hemocytes was determined in 10 µL of solution multiplied with dilution (Suljević et al. 2018). For the differential count, two drops of hemolymph were put onto the microscope slide for the preparation of the smear and waiting for spreading and was fixed for 5 min by using 99.8% methanol. The slide was turned at an angle of 45° to dry it at room temperature and stained by Giemsa stain for 20 min. The differentiation of hemocytes was determined at total count of 100 cells.

### Phagocytosis assay

The suspension of freshly prepared yeast cells was (0.1 gm of freshly obtained and dissolved in 10 mL PBS (pH 7.4 at 50 ºC in a water bath for 5 min in a glass test tube). The tube was transferred directly into ice to stop the reaction for 5 min. Then, it was centrifuged at 2000 g for 5 min and 3 times washing with cold PBS (Abdul-Salam and Michelson 1980). Freshly collected hemolymph (100 µL) was overlaid with an equal volume of yeast suspension on a clean glass slide. Then, incubation took place in a humid chamber for 60 min. The phagocytic the reaction was stopped using absolute methanol after washing with PBS (pH 7.4). The phagocytic index was calculated as a percentage of + ve hemocytes (+ ve, if it engulfed 1 or more yeast cells) from the calculated 100 cells/treatment.

### Statistical analysis

Data were expressed as the mean ± standard deviation and analyzed using the Statographics Centurion XVI (Stat-Point Technologies Inc., Warrenton, VA, USA). A two-way analysis of variance was used to identify differences between the control and exposed groups, between the exposed groups, and concentrations and durations. A probability *P* ≤ 0.05 level was accepted as significant.

## Results

### Toxicity of TiO_2_NPs

**Fig. 1:**
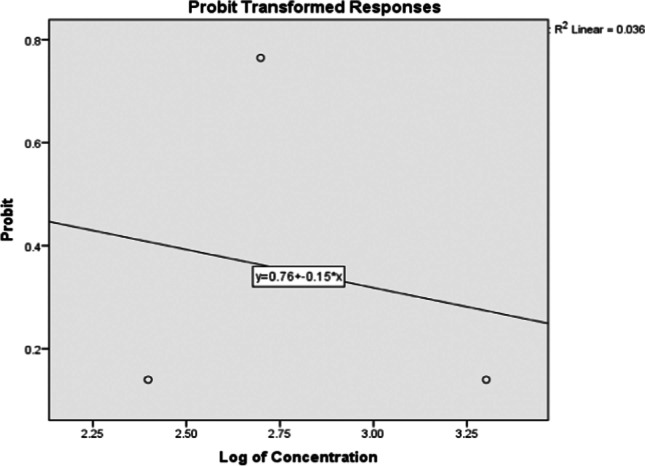
Toxicity of TiO_2_NPs against *Helix aspersa* after one day of exposure

The toxicity data of TiO_2_NPs against the snail *H. aspersa* showed that LC_50_ was 544 µg/L (95% confidence limit of log = 3.7) after 24 h of exposure (Fig. 1). The used sublethal two concentrations were 70 and 140 µg/L.

### Nanoparticle characterization

**Fig. 2:**
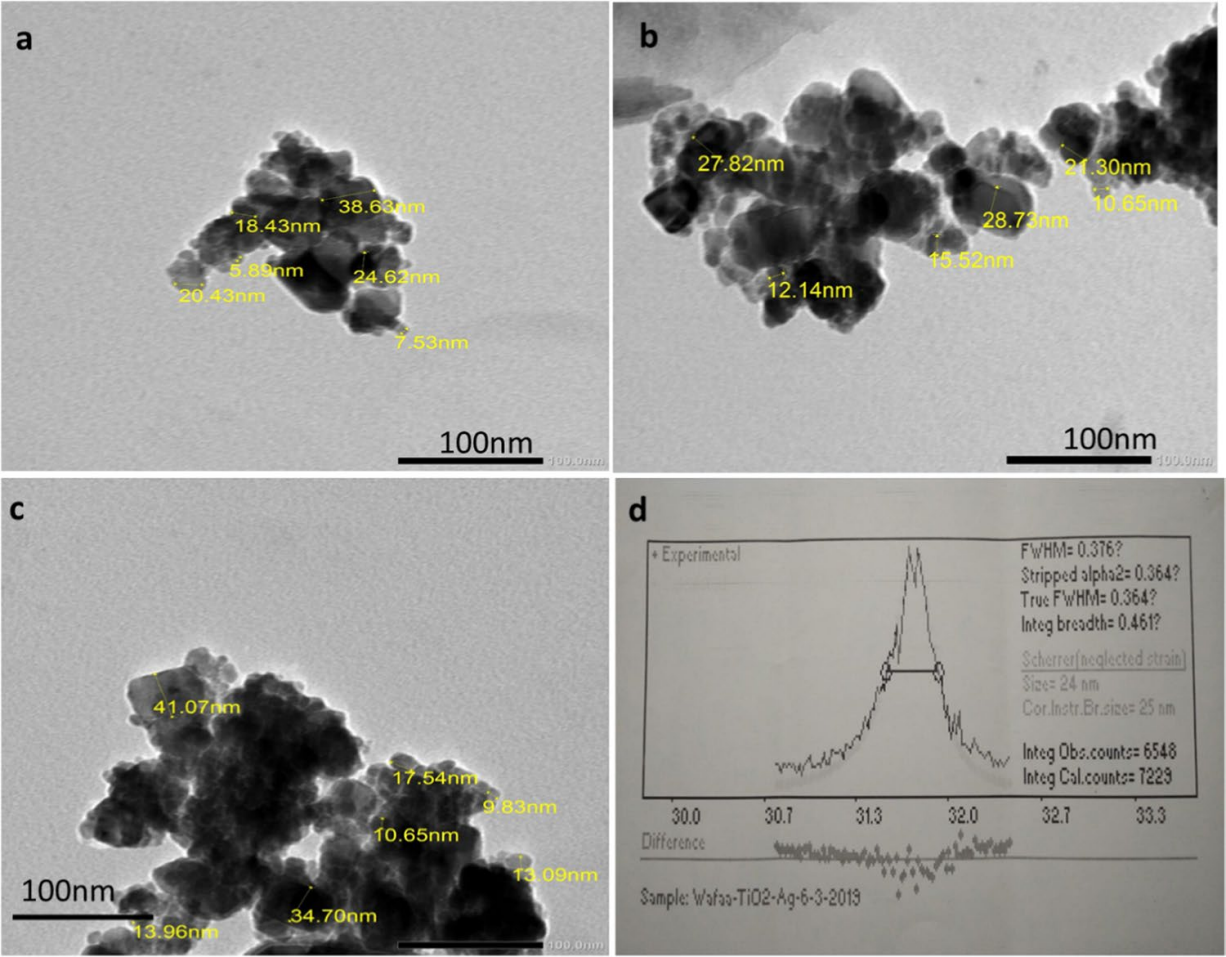
Characterization of TiO_2_NPs by transmission electron microscope (**a**–**c**) and XRD (**d**)

TEM image exhibited TiO_2_NPs as a characteristic spherical shape with sizes ranging from 7.5 to 41.00 nm as made-up in (Fig. 2a and b). The XRD pattern of TiO_2_ was illustrated in (Fig. 2c).

### Effect of TiO_2_NPs on condition index of the adult Helix aspersa (CI)

Condition index (CI) is a measure of the relative proportions of flesh-to-shell weight. TiO_2_NPs caused a reduction in the condition index by 10% when compared to the control (Table 1). At the end of the experiment, the value of CI of TiO_2_NP-exposed snails reached to 70.3% compared to that of the control 80%.

**Table 1:** Effect of TiO_2_NPs on the condition index (CI) of *Helix aspersa* after different time intervals

Time intervals	CI (%)		
	Control	70 µg/L	140 µg/L
Zero time	75	80	79.9
1 day	75	75	75
3 days	75	73.5	73
7 days	75	73	72
14 days	80	71	70.3*

Data showed as means, *n* = 5

^*^Represented significant difference between control and exposed groups when *P* ≤ 0.05

### Effect of TiO_2_NPs on the oxidative stress markers

**Fig. 3:**
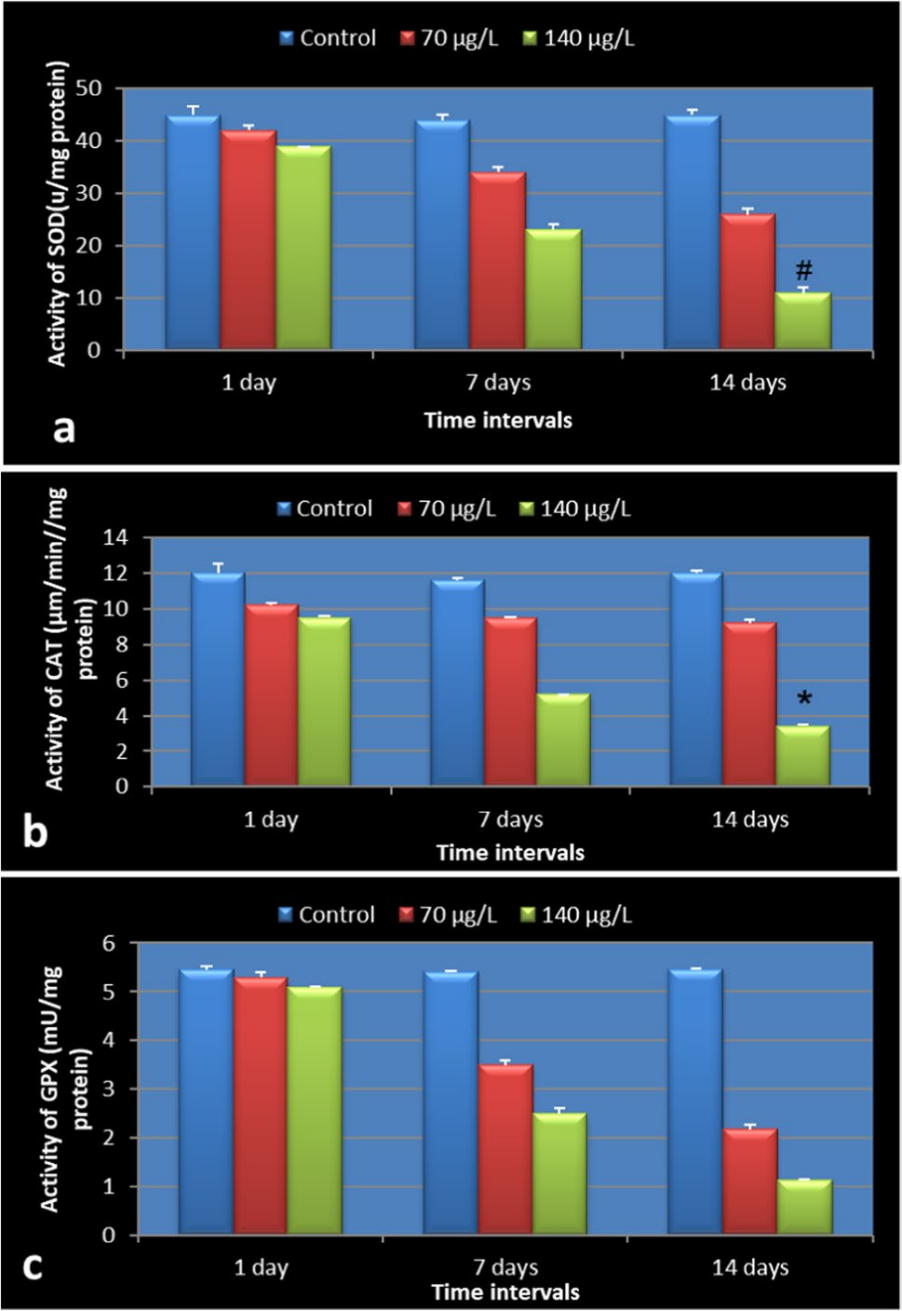
Effect of different concentrations of TiO_2_NPs on the activity of oxidative stress markers: **a** SOD, **b** CAT, and **c** GPX in the digestive gland of snail *Helix aspersa* after 14 days of exposure, values expressed as mean ± SD, significant difference indicated by (^*^) treatment vs. control (*P* = 0.0002), (^#^) vs. between concentration within durations (*P* = 0.001)

The exposure to TiO_2_NPs caused a reduction of the activities of SOD, CAT, and GPX in the digestive gland of the land snail *Helix aspersa* (Fig. 3a–c). The activity of catalase was reduced in both concentrations; the values were 9.5 and 5.2 µmole/min/mg protein at 70 µg/L and 140 µg/L, respectively, at seven days. The reduction was significant at the concentration of 140 µg/L after 14 days. That value was 3.4 µmole/min/mg protein (*P* = 0.001). SOD activity decreased in a concentration-dependent manner, but the significant reduction was at the concentration of 140 µg/L. The value was 11 µ/mg protein (*P* = 0.0002) at 14 days. Also, the concentration of 70 µg/L reduced the activity, and values were 34 and 26 µ/mg protein at seven days and 14 days, respectively, compared with the control 44 µ/mg protein (seven days) and 45 µ/mg protein (14 days). The activity of GPX reduced gradually till its value was 3.5 (seven days), 2.18 (14 days) at 70 µg/L compared to control 5.4 (seven days) and 5.47 (14 days) while 140 µg/L reduced the value to 1.13 mU/mg protein.

### Effect of TiO_2_NPs on DNA

**Fig. 4:**
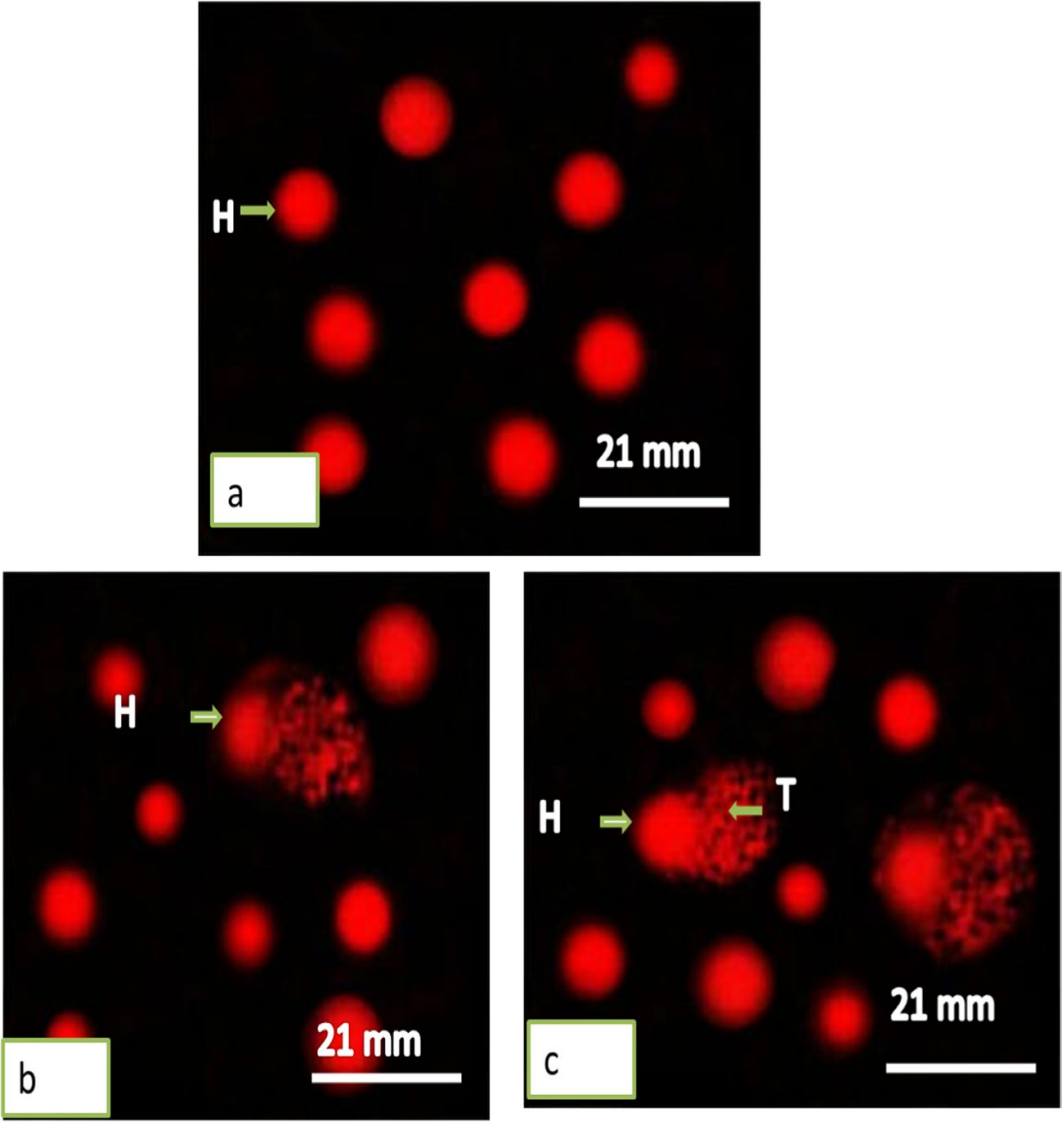
Photomicrograph showing DNA single-strand breaks (comet assay) in hemocytes of *Helix aspersa* snails exposed to two concentrations of TiO_2_NPs for 24 h. **a** Control (undamaged). **b** Treated with 70 µg/L. **c** Treated with 140 µg/L. H: head; T: tail

**Fig. 5:**
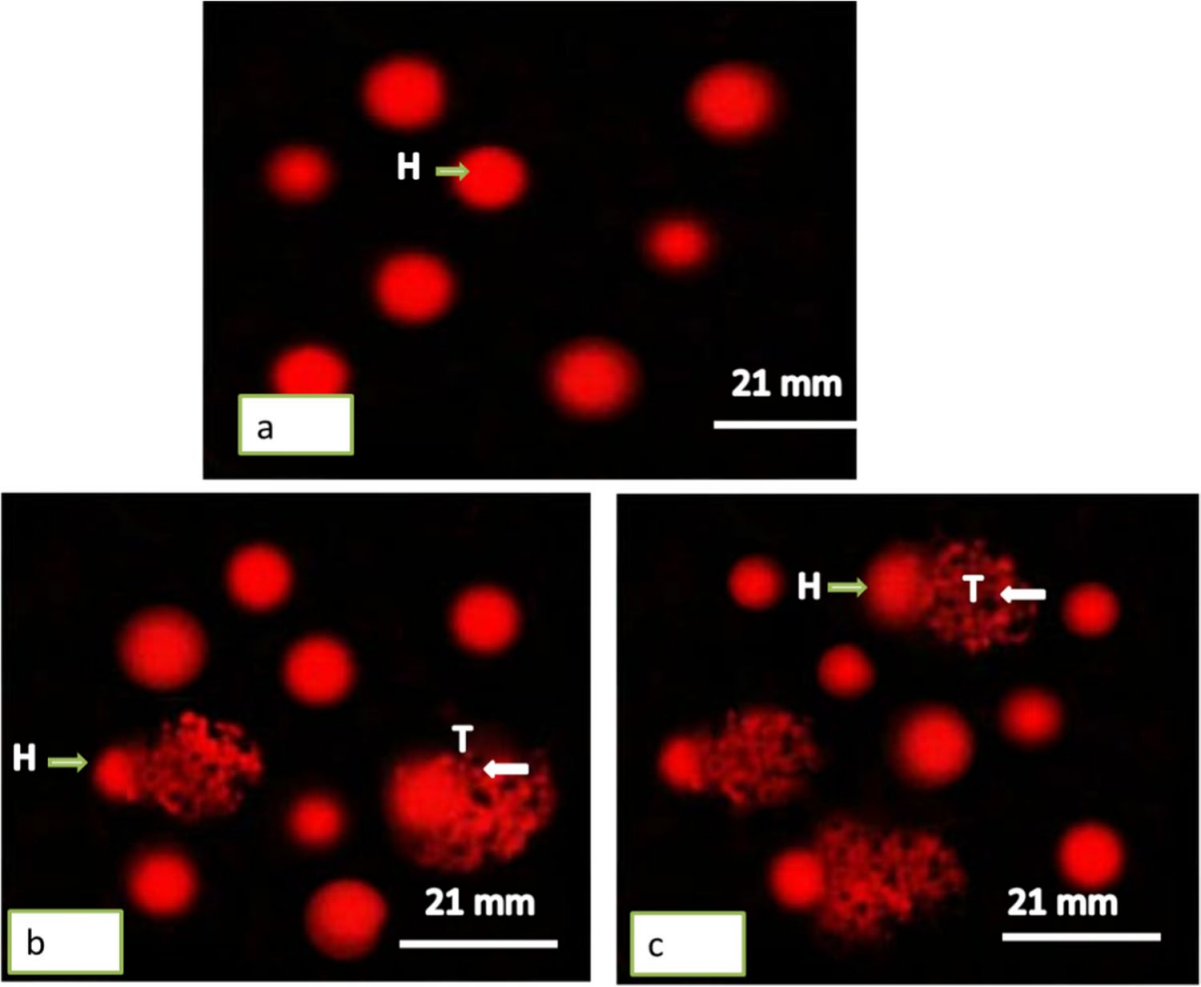
Photomicrograph showing DNA single-strand breaks (comet assay) in digestive gland of *Helix aspersa* snails exposed to two concentrations of TiO_2_NPs for 24 h. **a** Control (undamaged). **b** Treated with 70 µg/L. **c** Treated with 140 µg/L. H: head; T: tail

The DNA damage in hemocytes and the digestive gland of the land snail *Helix aspersa* after one day of exposure was represented in Tables 2 and 3 and Figs. 4 and 5. The treatment showed a significant increase (*P* ≤ 0.05) of DNA damage in the hemocytes of snails at the concentration of 140 µg/L. Then, the tail length was 6.12 ± 0.01 with 4-folds of increase compared with the controls (1.52 ± 0.01). The tail DNA showed the highest value (8.66 ± 0.02) with 5.3-folds increase. The significant increase in the tail moment was at the concentrations 140 µg/L (*P* = 0.002) with 21.37 folds of increase.

**Table 2:** Effect of TiO_2_NPs on DNA damage in the hemocytes of the snail *Helix aspersa* after one day of exposure

	Concentrations								
	Mean			% reduction			Fold of increase		
	Control	70 µg/L	140 µg/L	Control	70 µg/L	140 µg/L	Control	70 µg/L	140 µg/L
Tail length(µm)	1.52 ± 0.01	4.33 ± 0	6.12 ± 0.01*	–	–	–	–	2.8	4
Tail DNA (%)	1.63 ± 0.02	7.85 ± 0.02	8.66 ± 0.02	–			–	4.8	5.3
Tail moment	2.48 ± 0.02	33.99 ± 0.005	52.99 ± 0*	–			–	13.7	21.37
Tailed cell (%)	4 ± 0.1	12 ± 0.3	21 ± 1	–			–	3	5.25
Untailed (%)	96 ± 1	88 ± 0.5	79 ± 2.5	–	− 8.3	− 17.7	–	–	–

Data were expressed as mean ± S.D., *n* = 3

^*^Represented significant difference between control and exposed groups when *P* ≤ 0.05

**Table 3:** Effect of TiO_2_NPs on DNA damage in the digestive gland of the snail *Helix aspersa* after one day of exposure

	Concentrations								
	Mean			% reduction			Fold of increase		
	Control	70 µg/L	140 µg/L	Control	70 µg/L	140 µg/L	Control	70 µg/L	140 µg/L
Tail length(µm)	1.48 ± 0.01	6.25 ± 0.01	8.22 ± 0.11*#	–	–	–	–	4.2	5.5
Tail DNA(%)	1.55 ± 0.05	8.74 ± 0.04	10.63 ± 0.03	–	–	–	–	5.6	6.86
Tail moment	2.29 ± 0.09	51.38 ± 0.38	78.38 ± 0.08	–	–	–	–	22.37	34.23
Tailed cell (%)	8 ± 1	25 ± 3	36 ± 0	–	–	–	–	3.1	4.5
Untailed (%)	92 ± 1	75 ± 0.5	64 ± 1	–	− 18.48	− 30.43	–	–	–

Data were expressed as mean ± S.D., *n* = 3

^*^Represented significant difference between control and exposed groups ^**#**^Represented significant difference between the exposed groups when *P* ≤ 0.05

In the digestive gland, the tail length significantly increased at both concentrations, but the highest increase was at the concentration 140 µg/L (*P* = 0.0008) with 5.5-folds of increase. Both tail DNA and tail moment increased at concentrations in a dependent manner, but the highest value was in the tail moment (78.38 ± 0.08) with 34.23-folds of increase compared with controls (2.29 ± 0.09) (*P* = 0.0001).

### Effect of TiO_2_ nanoparticles on the hemocytes

The treatment with TiO_2_ nanoparticles significantly decreased the total amount of the hemocytes of *Helix aspersa* after two weeks. They were, respectively, compared with controls (79 ± 1.1 at 14 days) (Table 4).

**Table 4:** Effect of TiO_2_NPs on total hemocytes of snail *Helix aspersa* after two weeks of exposure

Treatments	Control	70 µg/L	140 µg/L
Time intervals			
24 h	80 ± 2	79 ± 1	78 ± 1
3 days	82 ± 1	78 ± 1.7	78 ± 1.1
7 days	80 ± 1.1	82 ± 5.2	75 ± 2
14 days	79 ± 1.1	74 ± 1	71 ± 1.5^*^

The number of hemocytes was determined in 10 µL. Data expressed as mean/10^4^ ± SD

^*^Significant at *P* ≤ 0.05

**Fig. 6:**
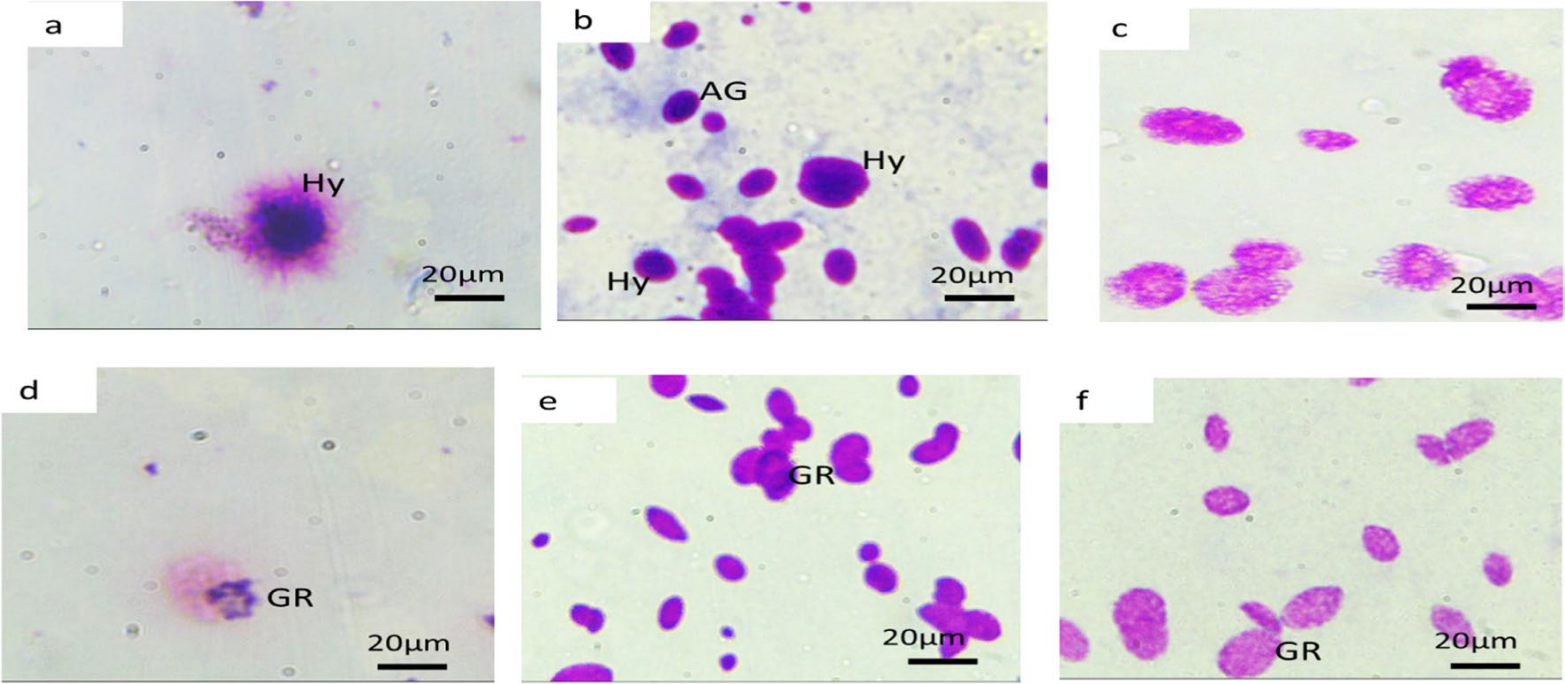
Light microscope of *H.aspersa* hemocytes. GR: granulocyte cell; Hy: hyalinocyte cell; AG: agranulocyte. **c** Transformed cell or immunocyte

Three types of hemocytes were distinguished: granulocytes represented by 55 ± 4%, characterized by numerous cytoplasmic granules, and form pseudopodia. Hylaniocytes are represented by 35 ± 2% and are characterized by a round shape with a large nucleus and their capacity to form filopodia. Agranulocytes were the smallest number of hemocytes. They have small round or elongated shapes. The cytoplasm had no granules (Fig. 6a, b, and d–f). There were numerous transformed hemocytes, which were named immunocytes. They resembled phagocytic cells and had numerous pseudopodia (Fig. 6c).

**Fig. 7:**
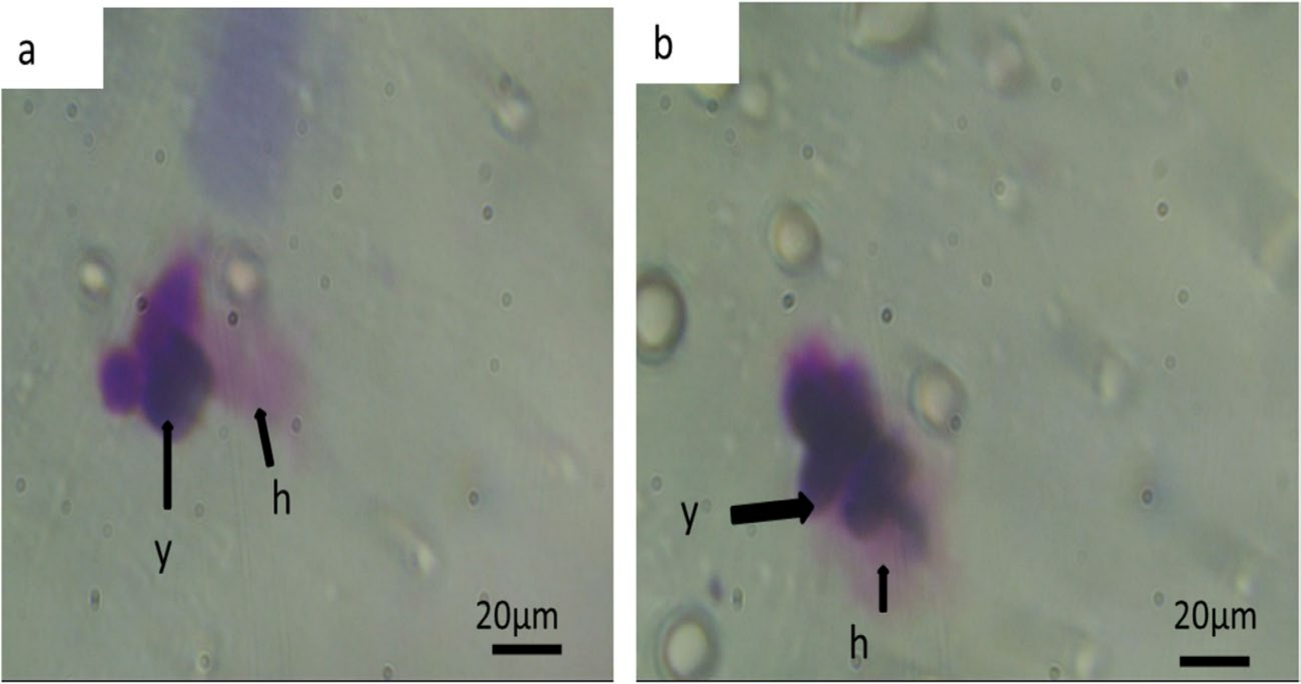
Light micrograph showing the phagocytic activity of hemocytes of exposed *Helix aspersa* after one day of exposure. h: hemocyte; y: yeast cells

The treatment increased the phagocytic activity. The percentage of phagocytosis after one day of exposure was 25 and 40% at 70 µg/L and 140 µg/L, respectively, compared with controls (19%) (Fig. 7).

### Histopathological alterations of the digestive gland of Helix aspersa exposed to TiO_2_NPs

**Fig. 8:**
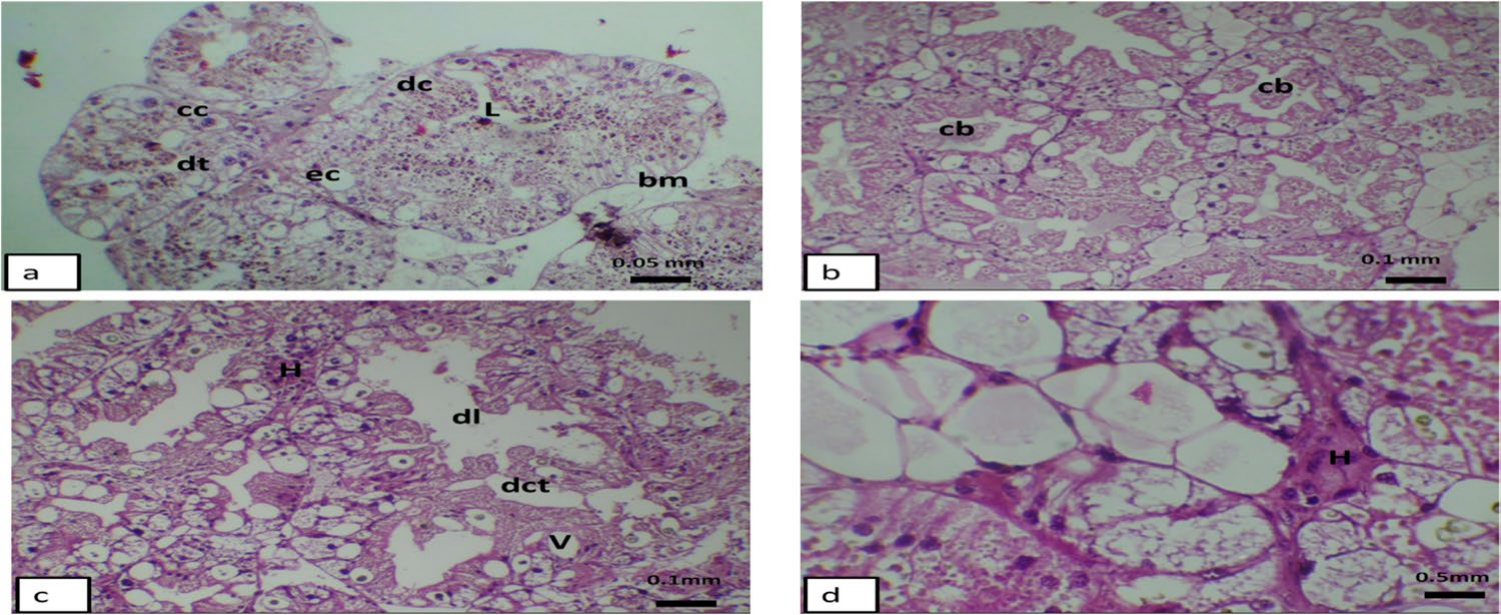
**a**–**d** Light micrograph showing effect of TiO_2_NPs at different concentrations on the digestive gland of snail *Helix aspersa* **a** control, digestive tubule (dt), digestive cell (dc), excretory cell (ec), basement membrane (bm), lumen (L). **b** Treated snails (70 μg/mL) showing presence of cellular blebs (cb) in lumen. (**c**, **d**) Treated snails (140 μg/mL) showing hemocytic infiltration (H), vacuoles (V), dilation of lumen (dl), and degenerated connective tissue (dct)

The microscopic examination of the digestive gland, or hepatopancreas of *Helix aspersa*, showed how it is vital in the visceral hump. It is composed of several digestive tubules, each one lined by simple epithelium containing different types of cells such as digestive, excretory, and calcium cells. They were arranged around a tight lumen (Fig. 8a). After treatment with 70 µg/L and 140 µg/L of TiO_2_ nanoparticles, some degeneration of the tubule, dilating of the lumen, and vacuolation appeared. Also, cellular blebs, which gave a sign of cell death, appeared. At the concentration of 140 µg/L, the tubule became more degenerated, and some inflammatory responses occurred as the hemocytic infiltration was obvious, such as the destruction of the tubule, enlarged lumen of the tubule, and the digestive cell turning completely necrotic with an increasing appearance of vacuoles (Fig. 8 b–d).

## Discussion

The toxicity of nanoparticles can be diagnosed by the determination of several biomarkers, such as oxidative stress markers and DNA damage. The imbalance in the biological antioxidant-to-oxidant ratio leads to oxidative damage causing damage to nucleic acids and proteins (Khene et al. 2017). The obtained results indicated that TiO_2_NPs caused a reduction of the activities of SOD, CAT, and GPX in the digestive gland of the land snail Helix *aspersa*. Decreased glutathione (GSH) content may be induced by free radicals produced by NPs or binding of glutathione to the metals (Barillet 2007). GPX is an enzyme that acts on the GSH content, which decreased as a result of the generation of free radicals produced by TiO_2_NPs (Abdel-Halim et al. 2021). The low GPX activity led to impaired antioxidant protection and, hence, caused oxidative damage to membrane functional proteins and fatty acids, besides neurotoxic damage (Chabory et al. 2009). In this context, Forgione et al. (2002) added that the depletion of GPX increased vascular oxidative stress. This finding was supported by many authors (Chandran et al. (2005) on the snail *Achatina fulica*, Khene et al. (2017) on *Helix aspersa*, and Ali et al. (2013, 2015, 2018, and ) on the snail *Lymnaea luteola*).

The activity of GPX got controlled by monitoring the generation of GSH from GSSG by the action of GR in presence of NADPH (Flohe and Gunzler 1984). Concerning the activity of CAT and SOD, CAT is a known antioxidant enzyme that uses the cofactor manganese and iron and stimulates hydrogen peroxide (H_2_O_2_) degradation. After that, SOD completed the detoxification process (Marklund 1984; Chelikani et al. 2004). The deficiency of SOD may be related to the deficiency of metals that it requires as cofactors are considered a metalloenzyme (Fridovich 1995; Dringen et al. 2005). Also, SOD deficiency may be related to the damage of the cells due to the treatment stated by Ighodaro and Akinloye (2018), which states that aging and cell death decline the level of SODs. On the contrary, Al-Abdan et al. (2021) found that sublethal concentrations of Bi_2_O3NPs significantly elevated SOD after 1, 3, and 7 days of exposure.

Chandran et al. (2005) recorded CAT inhibition in the hepatopancreas of the snail *Achatina fulica* after exposure to zinc and cadmium. Antioxidant enzyme activities depended on the exposure duration, concentration of pollutants, and species susceptibility (Ballesteros et al. 2009). The excessive production of reactive oxygen metabolites as a result of TiO_2_ nanoparticle treatment affected the cellular processes, especially membrane systems—hence, the cell viability. The autocatalysis of the oxidative damage process caused by high rates of free radicals’ input led to the reduction of enzymatic activities (Escobar et al. 1996). The decreased activities of antioxidant enzymes (SOD, CAT, and GPX) were noted by (Kono and Fridovich 1982) who reported that the excessive production of oxyradicals (O_2_ and OH) is attributed to TiO_2_ nanoparticle toxicity.

Generated ROS-induced DNA fragmentation so it can be used as a biomarker in snails against NPs (Reeves et al. 2008; Sidiropoulou et al. 2018; Kaloyianni et al. 2020). DNA fragmentation was explained by the fact that TiO_2_NPs penetrate the cells and enter the nucleus and snoop and bind to DNA nucleotides, inducing DNA breakage, thus leading to genotoxicity (Reeves et al. 2008). Boboria et al. (2020) found that exposure of the terrestrial land snail *Cornu aspersum* to titanium dioxide nanoparticles caused DNA damage, lipid peroxidation, protein carbonylation, and lysosomal membrane and apoptosis. Musee et al. (2010) and Croteau et al. (2011) reported malformations in nutrition, DNA damage, and oxidative stress as impacts of NPs on freshwater snails. Ali et al. (2015) diagnosed DNA fragmentation and decreased catalase activity in hemocyte cells of the freshwater snail *Lymnea luteola* L after treatment with different concentrations of TiO_2_NPs.

The digestive gland is considered the main organ involved in the detoxification of pollutants (Ismert et al. 2002). Several studies used the histological and histochemical changes in hepatopancreas as biomarkers of metal nanoparticle exposure (Manz et al. (2004) on *H. pomatia*; Souza Dahm et al. (2006) on *H. aspersa*).

Besnaci et al. (2016b) recorded the narrowing of the tubular lumen, degeneration, necrosis, and inflammation of the hepatopancreas of* Helix aspersa* after exposure to different doses of iron oxide nanoparticles.

The defense mechanisms in mollusks are sensitive to exposure to pollutants (Gagnaire et al. (2006) and Matozzo et al. (2005)). Also, the functions and density of molluscan hemocytes may become affected by some stressors such as xenobiotics (Livingstone et al. 2000; Galloway and Depledge (2001)). Ahmad (2010) characterized the granulocytes and hyalinocytes in the land snail *Helix aspersa*. Karuthapandi (2010) reported agranulocytes and granulocytes in the *Achatina fulica*. Suljević et al. (2018) reported the encapsulation ability of transformed hemocytes and, hence, its importance in phagocytosis.

## Conclusion

TiO_2_NPs affected the *Helix aspersa* in different aspects. The *Helix aspersa* is considered as an environmental pollution bioindicator. Hence, the release of TiO_2_NPs into the environment must be monitored.

## Data Availability

All data generated or analyzed during this study are included in this published article.
